# Phenotypic Impact and Multivariable Assessment of Antifungal Susceptibility in *Candida auris* Survival Using a *Galleria mellonella* Model

**DOI:** 10.3390/jof11060406

**Published:** 2025-05-24

**Authors:** Jorge Alvarruiz, Alba Cecilia Ruiz-Gaitán, Marta Dafne Cabanero-Navalon, Javier Pemán, Rosa Blanes-Hernández, Santiago de Cossio, Victor Garcia-Bustos

**Affiliations:** 1 Department of Internal Medicine, University and Polytechnic Hospital La Fe, 46026 Valencia, Spain; alvarruiz_jor@gva.es; 2 Severe Infection Research Group, Health Research Institute La Fe, 46026 Valencia, Spain; peman_jav@gva.es (J.P.); garcia_vicbus@gva.es (V.G.-B.); 3 Department of Microbiology, University and Polytechnic Hospital La Fe, 46026 Valencia, Spain; 4 Translational Research Group of Chronic Diseases and HIV Infection, University and Polytechnic Hospital La Fe, 46026 Valencia, Spain; blanes_ros@gva.es; 5 Unit of Infectious Diseases, University and Polytechnic Hospital La Fe, 46026 Valencia, Spain; decossio_san@gva.es; 6 Institute of Animal Health and Food Safety, University of Las Palmas de Gran Canaria, 35413 Arucas, Spain

**Keywords:** *Candida auris*, virulence, pathogenicity, aggregation, antifungal resistance, fitness tradeoffs, *Galleria mellonella*

## Abstract

The novel pathogen *Candida auris* has rapidly become a major health threat due to its high virulence, resistance to multiple antifungal agents, and remarkable environmental persistence. This study evaluated the influence of phenotypic traits and antifungal minimum inhibitory concentrations (MICs) on *C. auris* virulence using a *Galleria mellonella* infection model. Ten clinical strains, categorized as aggregative or non-aggregative, were analyzed for antifungal susceptibility and survival outcomes. All strains exhibited fluconazole resistance, with variable susceptibilities to other antifungals. Survival analysis revealed that the non-aggregative phenotype was independently associated with reduced survival in *G. mellonella* (HR = 2.418, *p* = 0.015), while antifungal MICs and invasive origin were not significant independent predictors of mortality in an elastic net-adjusted multivariable model. Strong correlations were observed between certain antifungal MICs, suggesting potential cross-resistance patterns; however, no independent association with virulence was identified. These results suggest that *C. auris* possesses not only an enhanced ability to develop antifungal resistance but also the capacity to do so without incurring fitness costs that could attenuate its virulence.

## 1. Introduction

Identified for the first time in 2009 from the ear canal of a patient in Japan [1], *Candida auris* has since been implicated in outbreaks across healthcare facilities worldwide [2,3,4,5]. Its rapid rise to prominence is attributable to a unique combination of traits that include multidrug resistance, high transmissibility, environmental resilience, and significant virulence [5,6,7,8,9]. The addition of such factors has conditioned the emergence of *C. auris* as a significant global health threat and raised substantial concerns within the medical and scientific communities [10,11].

*C. auris* is also characterized by a strikingly heterogeneous nature, with its strains classified into six distinct phylogenetic clades, each characterized by unique genetic profiles, antifungal susceptibility patterns, and epidemiological characteristics [6,12]. Furthermore, strains within the same clade can display significant variability in virulence and resistance traits. This complexity complicates efforts to draw generalized conclusions about biology and the pathogenesis of *C. auris*. Consequently, there is a pressing need to identify genetic and phenotypic patterns that correlate with the pathogen’s pathophysiological behavior, which could provide valuable insights to guide clinical decision-making [13,14].

Among the various factors contributing to the pathophysiology of *C. auris*, the aggregation phenotype and antifungal resistance have emerged as significant determinants of pathogenicity [5,8,15,16,17]. The aggregative phenotype, characterized by the formation of multicellular clumps, has been associated with altered interactions with the host immune system, distinct resistance profiles, and modulation of biofilm production [16,17,18]. Similarly, antifungal resistance is hypothesized to be a critical driver of *C. auris* virulence, as resistant strains often persist under therapeutic pressure, allowing for sustained infection and increased transmission [7,8,9]. However, antifungal resistance in pathogenic fungi can often be accompanied by fitness tradeoffs, which can reduce their virulence by impairing growth, biofilm formation, or other traits essential for host colonization and infection [19,20,21,22].

While multiple studies have found non-aggregative *C. auris* strains to exhibit higher virulence than their aggregative counterparts [16,17,18,23,24], the relationship between antifungal resistance and virulence in *C. auris* remains complex and incompletely understood. The investigation of this interplay could provide critical insights into the pathophysiology of *C. auris*, ultimately aiding in the risk stratification of infections and the optimization of therapeutic approaches. This study aims to investigate the virulence determinants of *C. auris* using a *G. mellonella* infection model, with a focus on exploring the relationships between antifungal susceptibility, minimum inhibitory concentrations (MICs), and their influence on pathogenicity and survival outcomes.

## 2. Materials and Methods

### 2.1. Fungal Strains

We randomly selected 10 *Candida auris* strains (124819, 182482, 253107, 312755, CJ98, CJ104, CJ173, CJ175, CJ197, and CJ198) from our institutional strain collection at the University and Polytechnic Hospital La Fe (UPHLF). These strains were originally isolated from both blood cultures and epidemiological surveillance samples collected from hospitalized patients. They were classified based on their origin as invasive or non-invasive strains. Patients from whom all three epidemiological surveillance non-invasive samples were obtained never developed candidemia or invasive disease.

#### 2.1.1. Blood Culture Processing and Strain Identification

Blood cultures were processed using the BacT/Alert Virtuo automated system (bioMérieux, Marcy l’Etoile, France). Initial identification of *C. auris* was performed in the Microbiology Department at UPHLF through sequencing of the internal transcribed spacer (ITS) region. Primers ITS3-ITS4 and ITS2-ITS5 were used with the GenomeLabTM GeXP system (Beckman Coulter, Fullerton, CA, USA). Confirmation was subsequently conducted at the Spanish Mycology Reference Laboratory employing ITS1-ITS4 primers.

#### 2.1.2. Phenotypic Classification

**Figure 1:**
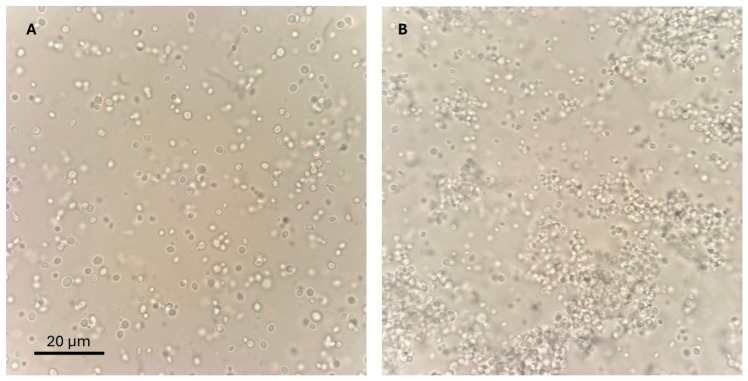
Representative micrographs of *Candida auris* phenotypes: (**A**) Non-aggregative phenotype displaying dispersed yeast cells with minimal clustering (CJ98). (**B**) Aggregative phenotype exhibiting large multicellular clusters, consistent with the classification criteria described by Borman et al., 2016 [17] (CJ198). 100× magnification. Scale bar: 20 μm.

Strains were phenotypically categorized into aggregative and non-aggregative groups. This was achieved by vortexing 1 mL of sterile saline, containing approximately 10^8^ CFU/mL, for 1 min. A 10 μL aliquot was then examined immediately under ×200 magnification using a TC20 automated cell counter (Bio-Rad Laboratories, Marnes-la-Coquette, France). The phenotype was classified as aggregative when large yeast clusters remained visible despite disaggregation procedures, as described by Borman et al., 2016 [17]. Figure 1 displays representative micrographs of both phenotypes observed in our strains under 100× magnification.

#### 2.1.3. Antifungal Susceptibility Testing

We assessed in vitro antifungal susceptibility following EUCAST standards [25]. The minimum inhibitory concentration (MIC) was defined as the drug concentration that inhibited 50% of fungal growth at 35 °C after 24 h (90% inhibition for amphotericin B). MIC values were determined according to the EUCAST methodology. However, interpretation of MIC values was performed using the tentative breakpoints established by the CDC for *C. auris*, given the current lack of EUCAST-specific breakpoints for this species at the moment of the experimental procedure.

### 2.2. Survival Assays in Galleria mellonella

#### 2.2.1. Larvae Handling

We obtained sixth-instar *Galleria mellonella* larvae from TruLarv (BioSystems Technology Ltd., Worcestershire, UK), a genome-sequenced breeding source. Only active larvae without melanization and weighing between 250 and 350 mg were selected. Initial surface decontamination was performed using 70% ethanol. The larvae were then grouped in sets of ten, placed in Petri dishes, and stored at 15 °C in the dark until they were ready for inoculation for a maximum of 72 h.

#### 2.2.2. Survival Assay Procedures

Survival experiments followed previously described methods [17]. Isolates of *C. auris* were cultivated on Sabouraud agar plates at 37 °C for 24 h. The resulting colonies were collected using sterile plastic loops, rinsed twice with sterile PBS, and quantified using a TC20 automated cell counter (Bio-Rad Laboratories, France). The final suspension was adjusted to 10^5^ CFU/μL. For *C. auris* aggregate strains, we prepared homogenous suspensions by allowing initial solutions to settle for 10 min, removing the supernatant with individual yeast cells, and then adjusting to 10^5^ CFU/μL, following the procedure detailed by Borman et al. (2016) [17]. The inoculum concentration (10^5^ CFU/μL, 10 μL per larva) was selected based on the reference method described by Borman et al., 2016 [17], to ensure standardization across experiments and comparability with prior *G. mellonella C. auris* infection models.

Larvae underwent a second decontamination with 70% ethanol prior to injection. Each larva received an intrahemocelic injection of 10 μL (containing 10^6^ CFU) into the left rear proleg using a 10 μL Hamilton syringe fitted with a 26-gauge blunt needle. Groups of 10–20 larvae per strain were injected with *C. auris* suspensions for the survival analysis. For each strain, an additional control group of 10 larvae injected with PBS was included. Only larvae that survived the first 12 h post-injection without signs of mechanical injury or early pupation were included in the final analysis.

Following injection, larvae were housed in groups of 10 in Petri dishes and incubated at 37 °C. Each dish was labeled with a unique identifier to ensure blinding during data collection. Mortality and any cocoon formation were recorded daily over a 10-day period, excluding deaths that occurred within the first 12 h post-inoculation.

### 2.3. Statistical Analysis

The statistical analysis was performed using R software, version 4.4.1.

Firstly, we assessed the relationships between phenotypes and MICs by calculating Spearman correlation coefficients. Spearman correlation was selected due to the categorical nature of the phenotype variable, which is appropriate for capturing monotonic relationships. Additionally, a correlation matrix using Pearson’s correlation coefficients was constructed to investigate potential correlations among different antifungal MICs. Correlations were computed using complete observations to ensure reliability. Variables showing strong correlations with an r coefficient > 0.8 were excluded in the survival analysis due to expected high multicollinearity. *p*-values were calculated with Bonferroni correction with a threshold of *p* < 0.05.

To assess the association between covariates and survival outcomes, we employed a two-step approach. Initially, a standard Cox proportional hazards regression was performed to estimate hazard ratios (HRs), coefficients, and *p*-values for each predictor variable, providing a preliminary evaluation of their associations with the outcome.

Subsequently, a Cox regression model with elastic net regularization was used to further refine the selection of relevant predictors, thereby identifying variables that significantly contributed to the model while managing multicollinearity and potential overfitting. The elastic net approach combines L1 (lasso) and L2 (ridge) penalties, allowing for the selection and shrinkage of coefficients, ultimately yielding a parsimonious model that highlights the most impactful variables. The elastic net model was fitted to optimize hyperparameters and enhance model generalizability. The final model was adjusted with the optimal lambda value, using an alpha value of 0.5 to balance between L1 (Lasso) and L2 (Ridge) regularization. The results of both models were compared to validate the robustness and relevance of the identified predictors.

Median survival times for each phenotype were estimated using the Kaplan–Meier method, and summary statistics of the survival analysis were presented to interpret survival differences across phenotypes.

## 3. Results

A total of 10 *C. auris* clinical strains were analyzed in this study, isolated from blood and non-invasive epidemiological surveillance samples obtained from hospitalized patients with diverse diagnoses across multiple departments. Non-invasive isolates were collected as part of a systematic weekly fungal surveillance and colonization monitoring program conducted in the surgical and medical ICUs of our institution. Based on phenotypic traits, three strains were classified as aggregative and seven as non-aggregative. Aggregative strains predominantly originated from polytraumatized patients in surgical ICUs, whereas non-aggregative strains were associated with a broader range of conditions, including febrile neutropenia, status epilepticus, and endocarditis (Table 1).

Antifungal susceptibility testing showed universal resistance to fluconazole (MIC > 256 mg/L) across all strains. Sensitivity to amphotericin B (MIC 0.25–2 mg/L) and echinocandins exhibited variability, with one strain being resistant to amphotericin B. Voriconazole MIC values were diverse, with 60% of isolates displaying MIC ≥ 2 mg/L; however, definitive resistance cannot be determined due to the absence of CDC-established breakpoints for voriconazole and other second-generation triazoles, for which fluconazole resistance serves as a surrogate indicator. Notably, non-aggregative strains demonstrated consistent susceptibility to caspofungin and anidulafungin (Table 2).

### 3.1. Correlation Analysis of Antifungal MICs Against C. auris: Exploring Associations and Managing Collinearity

The correlation matrix for the MICs of several antifungal agents against *C. auris* reveals notable associations (Table 3). Amphotericin B MIC shows strong correlations with anidulafungin (r = 0.912) and posaconazole (r = 0.938), indicating a strong positive relationship. Flucytosine MIC is also highly correlated with anidulafungin (r = 0.882) and caspofungin (r = 0.820) MICs, both considered strong correlations.

Itraconazole MIC also shows a strong correlation with flucytosine (r = 0.829) and a moderate correlation with caspofungin (r = 0.789). Voriconazole MIC shows a moderate correlation with micafungin (r = 0.712), suggesting a weaker yet notable association. Posaconazole MIC has a very high correlation with anidulafungin (r = 0.973), indicating an extremely strong positive relationship.

Only weak negative correlations were found between the non-aggregative and MICs to micafungin (r = −0.339), posaconazole (r = −0.363), and anidulafungin (r = −0.326). Negligible correlations were observed between the rest of the antifungal MICs.

### 3.2. Survival Assays in G. mellonella: Cox Regression and Elastic Net Analysis of Survival Predictors in a Control Model of C. auris Infection

Finally, 134 inoculated *G. mellonella* specimens completed in a valid manner the survival assays in the determined follow-up period. The total number of observed events was 134 for *C. auris*-infected larvae. No deaths or cocoon formation were observed in the control group; therefore, the data were extracted from the cohort and subsequent analyses and graphical representations. The median survival time was 2 days for the whole number of larvae. The initial cohort consisted of 134 individuals at risk, with a survival probability of 64.2% (95% CI: 56.6–72.8%) at 24 h. This probability progressively declined over time, reaching 32.1% (95% CI: 25.1–41.1%) at 48 h and further decreasing to 14.9% (95% CI: 10.0–22.4%) at 72 h. By day 9, survival probability was 0%, with no remaining individuals at risk.

Based on the correlation matrix, we identified high correlations between several variables, suggesting multicollinearity. To minimize this issue, we selected variables with lower correlations while keeping those that were clinically important. The final Cox model included phenotype, invasive origin, amphotericin B MIC, flucytosine MIC, voriconazole MIC, and micafungin MIC. This selection reduced multicollinearity and ensured that the model remained clinically meaningful.

The Cox proportional hazards model revealed that the non-aggregative phenotype of *C. auris* was significantly associated with reduced survival in *G. mellonella* (Hazard Ratio [HR] = 2.418, 95% CI: 1.190–4.913, *p* = 0.015). Invasive origin showed a trend toward an impact on survival, though it did not reach statistical significance (HR = 2.939, 95% CI: 0.830–10.41, *p* = 0.095). Further data can be seen in Table 4.

Antifungal MICs were not significantly associated with changes in survival. Amphotericin B MIC demonstrated an increased hazard ratio, indirectly related to that of flucytosine, although wide confidence intervals indicate considerable uncertainty about the true impact. Voriconazole MIC had a hazard ratio close to one, indicating minimal impact on survival. Micafungin MIC exhibited an extremely low hazard ratio. For all MICs, the wide confidence intervals reflect considerable uncertainty regarding their precise effects.

To address potential multicollinearity and adjust for variable selection, we subsequently conducted a Cox elastic net regression. The elastic net model confirmed the significant association of the non-aggregative phenotype with decreased survival (HR = 1.117). Variables such as invasive origin, amphotericin B MIC, flucytosine MIC, voriconazole MIC, and micafungin MIC were retained in the final model; however, none demonstrated a meaningful impact on survival (HR = 1.00 for all). This analysis suggests that the non-aggregative phenotype is an independent predictor of reduced survival, while the other variables did not provide additional explanatory power in the model.

**Figure 2:**
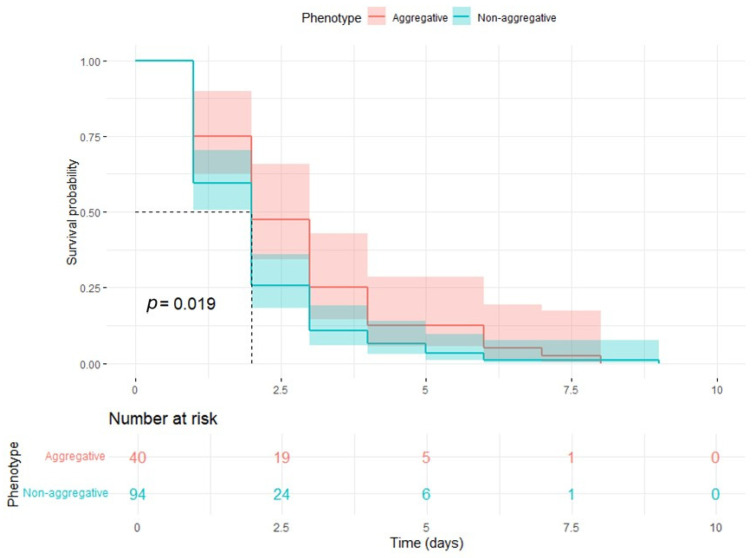
Kaplan–Meier survival curves of non-aggregative and aggregative *C. auris* phenotypes.

Considering that the phenotype was the only significant independent predictor of mortality, Kaplan–Meier survival curves were created, and survival statistics were calculated. In the survival analysis comparing aggregative and non-aggregative *C. auris* phenotypes, significantly distinct survival trends were observed (Figure 2) (*p* = 0.019). Aggregative phenotypes demonstrated higher initial survival probabilities, with 75% (95% CI: 62.7–89.7%) survival at time 1, decreasing to 25% (95% CI: 14.6–42.8%) at time 3, and 0% by time 8. Non-aggregative phenotypes exhibited lower survival probabilities from the outset, with 59.6% (95% CI: 50.4–70.4%) survival at time 1, declining to 10.6% (95% CI: 5.9–19.1%) at time 3, and 0% by time 9. Overall, non-aggregative phenotypes showed faster attrition in survival, reflected in steeper declines across time points, suggesting a potentially more severe clinical impact associated with this phenotype. Further data can be seen in Table 5 and Table 6.

## 4. Discussion

The main findings of this study can be summarized as follows: *C. auris* phenotype is an independent predictor of mortality in *G. mellonella* infection models, while other strain traits, such as susceptibility to antifungal drugs or the origin of the clinical isolates, have not been demonstrated to influence the virulence of *C. auris* infection in our regularized multivariable analysis in the in vivo model. Additionally, while all strains exhibited universal resistance to fluconazole, non-aggregative isolates showed broader antifungal susceptibility to echinocandins or posaconazole. Several strong positive correlations were found between different classes of antifungal drug MICs, suggesting different profiles of higher and lower antifungal susceptibility among the studied strains with potential cross-resistance mechanisms.

*Candida auris* is unique in many aspects of its pathophysiology. Its sudden and simultaneous emergence as a human pathogen on multiple continents, its high virulence, its environmental endurance, and high transmissibility, together with its increased tendency to develop antifungal resistance, justify the threat this microbe presents [5,7,8,9]. However, according to available data, not all strains of *C. auris* present the same virulence and resistance traits and mechanisms. Instead, substantial heterogeneity has been documented among the various *C. auris* clades and even between strains within the same clade [12,13,14]. It is hypothesized that *C. auris* possesses a remarkable capacity for genetic and phenotypic adaptation, and that this variability impacts its pathogenicity [13,14,26,27,28].

A known heterogenicity trait of *C. auris* is the capacity of some strains to grow forming aggregates [17]. Several studies have investigated the impact of this phenotype on fungal pathophysiology upon infection. Consistent with our findings, multiple authors have reported that non-aggregative strains exhibit greater virulence than aggregative strains, as demonstrated by the higher mortality rates observed in in vivo experiments [16,17,18,23]. However, *C. auris* has been shown to be able to form aggregates in mouse infection models regardless of phenotype [29], and Bing et al. retrieved aggregative colonies from the brain of their mouse infection model, which showed increased fitness in this tissue compared to non-aggregative strains [30]. Furthermore, differences between phenotypes in tissue tropism have been described in *G. mellonella* [16].

These aggregative strains are thought to have an advantage in colonization and environmental endurance [31]. Previous findings from our group indicate that less virulent aggregative strains exhibit a greater tendency to form pseudohyphae compared to non-aggregative strains, suggesting that aggregation and pseudofilamentation are more associated with adaptation than pathogenicity [16]. Additionally, Short et al. reported that aggregative *C. auris* strains exhibit greater medium survivability and enhanced resistance to sodium hypochlorite [32]. However, these traits may also depend on environmental and host conditions, as Brown et al. observed increased virulence of aggregative *C. auris* strains in a skin infection model [33].

Decreased antifungal susceptibility is also attributed to the aggregative phenotype [34]. Though contradictory results have been found [23], various authors have reported increased biomass of biofilm produced by aggregative strains in comparison with non-aggregative isolates [24,28,31,32]. In addition to this, in line with our results, other authors have also described higher MICs for aggregative less-virulent strains [32,34]. Furthermore, Louvet et al. identified genetic mechanisms that promote aggregation, enhance biofilm production, and increase surface adhesion [28]. Finally, several studies have documented the formation of aggregates in *C. auris* colonies following antifungal exposure [35,36,37,38]. Collectively, these findings suggest reduced susceptibility of the aggregative phenotype to certain antifungal drugs and support the associations observed in our results between phenotype and MIC values.

Antifungal susceptibility is one of the main concerns in the management of *C. auris* infection and colonization due to the tendency of this pathogen to rapidly develop resistance. Historically, the acquisition of antibiotic resistance has been associated with fitness trade-offs due to the evolutionary pressure exerted by the drug, often resulting in less virulent resistant strains. This phenomenon has been extensively documented in *Candida* species [19,20,21,22], though exceptions to this rule have also been reported [39,40,41,42]. In the case of *C. auris*, resistant strains have sometimes shown reduced virulence compared to their susceptible counterparts [35,38,43,44,45]. However, Burrack et al. observed no loss of fitness in resistant *C. auris* strains, as evidenced by the stability of resistance-conferring mutations even in the absence of antifungal drugs [15]. Similarly, Bohner et al. evolved *C. auris* strains to acquire triazole resistance and assessed their virulence in a murine model. While some resistant strains exhibited hypovirulence, with lower fungal burdens in mouse tissues, others surpassed their parental strains in their ability to colonize brain tissue [46]. Additionally, Carolus et al. reported decreased growth and stress resistance in resistant *C. auris* strains but demonstrated that these deficits could be reversed through compensatory mutations introduced via molecular engineering [43]. Consistent with these findings, our study found no correlation between antifungal MICs, independently of resistance breakpoints, and virulence, suggesting that *C. auris* can acquire antifungal resistance without incurring significant fitness costs. The reviewed data highlight the substantial variability in *C. auris* antifungal susceptibility profiles and suggest the existence of heterogeneous resistance mechanisms, which may underlie the pathogen’s remarkable ability to develop resistance to all currently available antifungal drugs.

Finally, the MIC correlation patterns observed across antifungal classes in *C. auris* are biologically plausible and reflect this pathogen’s propensity for multidrug resistance. Notably, we found that amphotericin B MICs strongly correlate with anidulafungin MICs (r ≈ 0.91) and posaconazole MICs (r ≈ 0.94), and similarly high correlations exist between azoles and echinocandins (e.g., posaconazole vs. anidulafungin, r ≈ 0.97) and between flucytosine and echinocandins (flucytosine vs. anidulafungin, r ≈ 0.88). At first glance, such cross-class associations seem counterintuitive given the distinct targets of these drugs—amphotericin B binds ergosterol in the fungal membrane, azoles inhibit ergosterol biosynthesis, echinocandins block β-1,3-glucan cell wall synthesis, and flucytosine disrupts DNA/RNA synthesis. Direct cross-resistance between these pathways is not classically expected, as correlations between minimum inhibitory concentrations of azoles and echinocandins, amphotericin and echinocandins, and flucytosine and echinocandins in *C. auris* are not well-established and are influenced by specific genetic mutations and resistance mechanisms unique to each antifungal class. However, *C. auris* frequently harbors broad resistance mechanisms that elevate MICs across multiple antifungal classes [47,48]. One of the best documented and understood instances is the appearance of cross-resistance between azoles and polyenes due to EGR gene mutations, as both of these drugs exercise their antifungal function by interfering with ergosterol physiology, which is closely regulated by said genes [43,49,50]. However, other associations have been described without a clear mechanism. For example, exposure to echinocandins can select for mutations in the ergosterol pathway (ERG3) that confer cross-resistance to azoles [47]. Likewise, alterations in cell membrane composition that reduce amphotericin B binding (e.g., via ERG gene mutations) could coincidentally affect cell wall integrity or drug uptake, increasing echinocandin MICs as well [47]. Indeed, *C. auris* isolates with simultaneous resistance to azoles, polyenes, echinocandins, and even 5-flucytosine, have been documented [48,51], often through the accumulation of multiple resistance determinants (such as ERG11 mutations for azoles, FKS1 hot-spot mutations for echinocandins, ERG3 or ERG6 alterations for amphotericin B, and FUR1 loss-of-function for flucytosine). Furthermore, our data and others suggest that certain phenotypes (e.g., the aggregative phenotype in *C. auris*) inherently exhibit elevated MICs to a broad range of antifungals, which would naturally produce positive inter-MIC correlations. In light of these factors, it is logical that isolates with high MICs to one drug class often show high MICs to others—not necessarily due to direct target cross-resistance, but due to co-occurring resistance mechanisms and global stress-response adaptations. This co-tolerance underscores the clinical challenge posed by *C. auris*: resistance to one antifungal agent frequently does not occur in isolation but is part of a multidrug-resistant phenotype [47]. The strong amphotericin B–anidulafungin and azole–echinocandin MIC correlations observed are therefore consistent with known resistance patterns in *C. auris*, and they highlight the need for vigilance as resistance in this organism can span disparate drug classes.

Despite the robustness of the two-step statistical analysis, which included a multivariable approach using Cox regression with elastic net regularization, several limitations should be acknowledged. On the one hand, the number of strains analyzed was relatively small. Given the heterogeneous nature of the species, a more comprehensive study involving a larger number of isolates and a broader representation of clades, clinical origins, and phenotypes would be valuable. Moreover, although a standardized inoculum of 10⁶ CFU per larva was used based on validated protocols, future studies evaluating a broader range of inocula (e.g., 10^3^ to 10^8^ CFU) could help delineate dose-dependent virulence patterns and assess the potential influence of quorum-sensing mechanisms in *C. auris* pathogenesis. The absence of a standardized reference *C. auris* strain could represent a limitation of this study for comparability. However, it is important to note that, to date, there are no universally defined reference strains for the aggregative and non-aggregative phenotypes of *C. auris*. Solidly establishing these reference models would greatly enhance comparative analyses in future studies. On the other hand, as this study employed an exploratory multivariable virulence animal model, key fungal virulence factors—such as biofilm production, filamentation, and enzyme activity—were not explicitly evaluated. Incorporating genomic or proteomic analyses could provide further insights into the pathophysiology of *C. auris* during infection. Additionally, this study did not include specific in vitro assays for individual virulence traits such as growth rate, thermotolerance, lipid membrane composition, biofilm formation, or hyphal development. Future studies incorporating these other phenotypic evaluations or genomic determinants are warranted to better explain the mechanisms underlying *C. auris* pathogenicity and antifungal resistance, and to deepen the understanding of the phenotypic variability observed across strains

## 5. Conclusions

A major challenge in studying and managing this pathogen is the pronounced genetic and phenotypic heterogeneity among its strains. One such phenotypic trait is the formation of aggregates, which, according to our findings and supporting evidence, impacts both virulence and antifungal susceptibility. Non-aggregative strains appear to exhibit increased virulence in invasive infections, whereas the aggregative phenotype seems to enhance colonization through improved environmental survivability and antifungal resistance. Higher MICs to antifungal drugs, however, do not seem to be independent predictors of mortality in in vivo animal models of infection. Further phenotypic, molecular, and genomic characterization of the differences between *C. auris* phenotypes could deepen our understanding of its pathophysiology and potentially provide useful markers for the clinical management of both infection and colonization.

## Figures and Tables

**Table 1:** Basic characteristics of *C. auris* strains used in the study according to isolate origin and phenotype. ICU, intensive care unit.

Time	Number at Risk	Number of Events	Survival Probability	Standard Error
Invasive samples				
CJ104	Blood	Polytraumatized	Surgical ICU	Aggregative
CJ173	Blood	Polytraumatized	Surgical ICU	Aggregative
Cj198	Blood	Pneumonia	Medical ICU	Aggregative
CJ98	Blood	Polytraumatized	Surgical ICU	Non-aggregative
CJ175	Blood	Status epilepticus	Medical ICU	Non-aggregative
CJ197	Blood	Febrile neutropenia	Hematology	Non-aggregative
312775	Blood	Endocarditis	Medical ICU	Non-aggregative
Non-invasive epidemiological surveillance samples				
124819	Rectal	Extracorporeal mechanical oxygenation	Medical ICU	Non-aggregative
182482	Inguinal	Liver transplantation	Medical ICU	Non-aggregative
253107	Pharyngeal	Multiple myeloma	Medical ICU	Non-aggregative

**Table 2:** Data on antifungal susceptibility and MIC values of *C. auris* strains used in the study according to aggregative and non-aggregative phenotypes. MIC, minimum inhibitory concentration; AMB, amphotericin B; 5FC, flucytosine; FLU, fluconazole; ITR, itraconazole; VOR, voriconazole; POS, posaconazole; CAS, caspofungin; ANI, anidulafungin; MCF, micafungin; S, susceptible; R, resistant; IE, insufficient evidence.

		MIC (mg/L)																	
Strain	Phenotype	AMB MIC	AMB	5FC MIC	5FC	FLU MIC	FLU	ITR MIC	ITR	VOR MIC	VOR	POS MIC	POS	CAS MIC	CAS	ANI MIC	ANI	MCF MIC	MCF
124819	Non-aggregative	0.5	S	<0.06	S	>256	R	0.06	IE	0.03	IE	0.015	IE	0.03	S	0.06	S	0.03	S
182482	Non-aggregative	0.5	S	<0.06	S	>256	R	0.06	IE	0.03	IE	0.015	IE	0.03	S	0.03	S	0.03	S
253107	Non-aggregative	0.5	S	<0.06	S	>256	R	0.06	IE	0.03	IE	0.015	IE	0.03	S	0.125	S	0.03	S
CJ98	Non-aggregative	0.25	S	0.12	S	>256	R	0.25	IE	2	IE	0.03	IE	0.5	S	0.06	S	0.06	S
CJ175	Non-aggregative	0.5	S	0.06	S	>256	R	0.125	IE	2	IE	0.06	IE	0.03	S	0.125	S	0.06	S
CJ197	Non-aggregative	2	R	0.25	S	>256	R	0.25	IE	4	IE	0.06	IE	0.5	S	0.5	S	0.25	S
312775	Non-aggregative	0.5	S	0.06	S	>256	R	0.125	IE	8	IE	0.06	IE	0.03	S	0.125	S	0.06	S
CJ104	Aggregative	0.5	S	0.06	S	>256	R	0.125	IE	2	IE	0.06	IE	0.03	S	0.125	S	0.06	S
CJ173	Aggregative	0.5	S	<0.06	S	>256	R	0.06	IE	2	IE	0.03	IE	0.06	S	0.06	S	0.06	S
CJ198	Aggregative	0.25	S	0.06	S	>256	R	0.25	IE	1	IE	0.03	IE	0.06	S	0.125	S	0.06	S

**Table 3:** Correlation matrix of minimum inhibitory concentration (MIC) values against tested antifungals in *C. auris* isolates: Pearson test for inter-MIC correlations and Spearman test for MIC-phenotype associations. Bold represents values related to more than >80% correlation. *, *p*-value below 0.05 with Bonferroni correction.

	Amphotericin B MIC	Flucytosine MIC	Itraconazole MIC	Voriconazole MIC	Caspofungin MIC	Anidulafungin MIC	Micafungin MIC	Posaconazole MIC
Amphotericin B MIC	1.000	0.659 *	0.191	0.297 *	0.346 *	**0.912** *	0.372 *	**0.938** *
Flucytosine MIC	0.659 *	1.000	**0.829** *	0.490 *	**0.820** *	**0.882** *	0.558 *	**0.815** *
Itraconazole MIC	0.191	**0.829** *	1.000	0.340 *	0.789 *	0.551 *	0.357 *	0.458 *
Voriconazole MIC	0.297 *	0.490 *	0.340 *	1.000	0.226	0.445 *	0.712 *	0.428 *
Caspofungin MIC	0.346 *	**0.820** *	0.789 *	0.226	1.000	0.587 *	0.108 *	0.427
Anidulafungin MIC	**0.912** *	**0.882** *	0.551 *	0.445 *	0.587 *	1.000	0.526 *	**0.973** *
Micafungin MIC	0.372 *	0.558 *	0.357 *	0.712 *	0.108 *	0.526 *	1.000	0.560 *
Posaconazole MIC	**0.938** *	**0.815** *	0.458 *	0.428 *	0.427	**0.973** *	0.560 *	1.000
Phenotype	0.116	0.009	−0.101	−0.167	−0.020	−0.326	−0.339	−0.363

**Table 4:** Summary of Cox proportional hazards regression analysis for factors affecting survival in the *G. mellonella C. auris* infection model. *: statistical significance below 0.05.

Variable	Coefficient	Hazard Ratio	Lower 95% Confidence Interval	Upper 95% Confidence Interval	Z-Score	*p*-Value
Non-aggregative phenotype	0.883	2.418	1.190	4.913	2.441	0.015 *
Invasive origin	1.078	2.939	0.830	10.41	1.671	0.095
Amphotericin B MIC	1.038	2.823	0.1661	47.96	0.718	0.473
Flucytosine MIC	−0.991	0.371	3.7 × 10^−6^	3.72 × 10^4^	−0.169	0.866
Voriconazole MIC	−0.116	0.891	0.771	1.029	−1.571	0.116
Micafungin MIC	−9.872	5.16 × 10^−5^	1 × 10^−18^	2.6 × 10^9^	−0.613	0.540

**Table 5:** Survival analysis in aggregative *C. auris* phenotypes. NA: not available.

Time	Number at Risk	Number of Events	Survival Probability	Standard Error	Lower 95% CI	Upper 95% CI
1	40	10	0.75	0.0685	0.62713	0.897
2	30	11	0.475	0.079	0.34293	0.658
3	19	9	0.25	0.0685	0.14616	0.428
4	10	5	0.125	0.0523	0.05506	0.284
6	5	3	0.05	0.0345	0.01295	0.193
7	2	1	0.025	0.0247	0.00361	0.173
8	1	1	0.0	NA	NA	NA

**Table 6:** Survival analysis in non-aggregative *C. auris* phenotypes. NA: not available.

Time	Number at Risk	Number of Events	Survival Probability	Standard Error	Lower 95% CI	Upper 95% CI
1	94	38	0.5957	0.0506	0.50436	0.7037
2	56	32	0.2553	0.045	0.18078	0.3606
3	24	14	0.1064	0.0318	0.05921	0.1911
4	10	4	0.0638	0.0252	0.02943	0.1384
5	6	3	0.0319	0.0181	0.01048	0.0972
6	3	2	0.0106	0.0106	0.00151	0.0747
9	1	1	0.0	NA	NA	NA

## Data Availability

The raw data supporting the conclusions of this article will be made available by the authors on request.
